# Whole-Genome Sequence of Pseudomonas aeruginosa Strain 4014, Isolated from Soil in France

**DOI:** 10.1128/MRA.01089-18

**Published:** 2018-09-13

**Authors:** Julien Crovadore, Damien Grizard, Romain Chablais, Bastien Cochard, Philippe Blanc, François Lefort

**Affiliations:** a Plants and Pathogens Group, Research Institute Land Nature and Environment, hepia, HES-SO University of Applied Sciences and Arts Western Switzerland, Jussy, Geneva, Switzerland; b Cybèle Agrocare, Levallois-Perret, France; c Bioprox, Noyant, France; Louisiana State University

## Abstract

We report here the draft genome sequence of strain 4014 of Pseudomonas aeruginosa, a common human pathogen, isolated from soil in France. This sequence predicts resistance to multiple antibiotics, including vancomycin.

## ANNOUNCEMENT

Pseudomonas aeruginosa (Schroeter 1872) Migula 1900 is a Gram-negative rod-shaped gammaproteobacterium, which is motile through a single polar flagellum and produces a unique water-soluble blue-green pigment called pyocyanin (1). This bacterium is a resident of terrestrial and aquatic environments and may be found in plants, animals, and humans (2–4). An opportunistic human pathogen, it is a major cause of lethal nosocomial infections by reason of its resistance to many antibiotic classes (5–7).

Isolated from soil in western France, on King B agar, strain 4014 of Pseudomonas aeruginosa was initially identified as Pseudomonas otitis by biochemical phenotyping. DNA was extracted, according to a modified cetyltrimethylammonium bromide (CTAB) protocol (8), from a culture grown to its exponential phase in King B broth. Sequencing of the 16S rRNA gene showed that this strain shared 100% identity with more than 100 Pseudomonas aeruginosa strains of the GenBank 16S database. A library was created with the TruSeq Nano DNA library preparation kit (Illumina, USA). The shotgun sequencing was performed in one Illumina MiniSeq run at a 2 × 151-bp paired-end read length, with a MiniSeq high-output kit, resulting in a 259× genome coverage.

Once quality control was carried out with FastQC version 0.11.5 (9), genome assembly was performed with SPAdes genome assembler 3.10 (10), with settings fixed in “paired-end assembly, careful mode,” yielding 131 contigs (≥200 bp), which were then arranged with BioEdit version 7.0.5 (11) and analyzed with QUAST version 4.6.3 (12), with the setting “QUAST: skip contigs shorter than 200 bp.” The genome's total length was 6,518,993 bp, with a GC content of 66.01% and an *N*_50_ value of 150,161 bp. Automated gene annotation was carried out by the NCBI Prokaryotic Genome Annotation Pipeline (PGAP) version 4.1 (13) and RAST version 2.0 (14). PlasmidFinder version 1.3 (15), with default settings, and plasmidSPAdes (16), with default settings, did not detect any plasmid. No complete phage sequence was retrieved. RAST, using the ClassicRAST annotation scheme, identified 6,041 coding sequences and 67 RNA genes, while PGAP retrieved 6,324 genes and 70 RNA sequences. No known toxin or virulence genes and no known pathogenicity islands were detected. The nitrogen metabolism accounted for 89 genes, among which were 1 nitrilase, 5 nitrate reductase, and 2 nitrite reductase genes. Four genes might be related to auxin synthesis. More than 50 genes are involved in siderophore sensing, biosynthesis, transport, and reception, with pyoverdin and pyochelin being the main siderophores. This strain harbors 36 genes for complete type III secretion systems organized in a large operon on contig 127 and 41 genes for a type VI secretion system, which is the virulence factor for Pseudomonas aeruginosa (17). Phenazine biosynthesis, which is essential to pyocyanin production (18), is encoded by 14 genes. Degradation of aromatic compounds is encoded by 122 genes. This bacterium is equipped to resist antibiotics and metals (133 genes). This annotation predicts resistance to vancomycin, fosfomycin, fluoroquinolones, and beta-lactam antibiotics. Motility and chemotaxis are encoded by 116 genes. This genome resource adds to the knowledge of the species Pseudomonas aeruginosa.

### Data availability.

This whole-genome shotgun (WGS) project was deposited at DDBJ/EMBL/GenBank under the accession number NFRZ00000000. The version described in this paper is the first version, NFRZ01000000. The 131 contigs have been deposited under the accession numbers NFRZ01000001 through NFRZ01000131. Raw sequencing data sets have been registered in the NCBI Sequence Read Archive database (19) under the accession number SRR5513015.
